# Systemic postnatal corticosteroids and magnetic resonance imaging measurements of corpus callosum and cerebellum of extremely preterm infants

**DOI:** 10.1111/jpc.16286

**Published:** 2022-11-20

**Authors:** Charmaine Han‐Menz, Gillian Whiteley, Rachel Evans, Abdul Razak, Atul Malhotra

**Affiliations:** ^1^ Department of Paediatrics Monash University Melbourne Victoria Australia; ^2^ Diagnostic Imaging Monash Health Melbourne Victoria Australia; ^3^ Monash Newborn Monash Children's Hospital Melbourne Victoria Australia; ^4^ Ritchie Centre Hudson Institute of Medical Research Melbourne Victoria Australia

**Keywords:** cerebellum, corpus callosum, extremely premature, magnetic resonance imaging, steroids

## Abstract

**Aim:**

To compare the size of the corpus callosum (CC) and cerebellum on magnetic resonance imaging (MRI) brain scans conducted at term equivalent age (TEA) in extremely preterm infants who received systemic postnatal corticosteroids (PCS) to extremely preterm infants who did not receive systemic PCS and determine the dose‐dependent effects on these outcomes.

**Methods:**

Single‐centre retrospective cohort study including extremely preterm infants (born < 26 weeks' gestation) who had MRI brain scans at TEA. CC and cerebellar measurements were evaluated by two radiologists who were blinded to steroid use and their independent measurements were averaged. Comparative analyses were conducted between exposed (to systemic PCS) and non‐exposed groups.

**Results:**

Eighty‐three extremely preterm infants with mean (SD) 24.9 (0.91) weeks' gestational age, 721.8 (156) g birthweight were included; 38 with systemic PCS exposure and 45 without exposure. After adjustment for birthweight and other significant neonatal morbidities, there was no significant difference noted in corpus callosum length (CCL) between unexposed and exposed groups (adjusted mean (SE) 39.5 (0.57) mm vs. 38.5 (0.62) mm; *P* = 0.29). Similarly, the ratios of CCL/fronto‐occipital diameter (FOD) and CCL/biparietal diameter (BPD) were not significantly different between the groups (CCL/FOD (0.40 (0.01) vs. 0.41 (0.01); *P* = 0.70) and CCL/BPD (0.51 (0.01) vs. 0.52 (0.01); *P* = 0.62)). Finally, no significant differences in cerebellar measurements, such as vermian height (adjusted mean (SE) 24.0 (0.46) mm vs. 23.5 (0.51 mm); *P* = 0.47) and transcerebellar diameter (adjusted mean (SE) 49.3 (0.74) mm vs. 4.78 (0.82) mm; *P* = 0.22) were found. No dose‐dependent effects of systemic PCS on CC and cerebellar measurements were identified.

**Conclusions:**

Systemic PCS use in extremely preterm infants was not associated with a change in the CC and cerebellar measurements on MRI brain scan at TEA.

## What is already known on this topic

1. Systemic postnatal corticosteroids are commonly used in extremely preterm infants.
2. Postnatal corticosteroid use has been associated with adverse neurodevelopmental outcomes.
3. There is paucity of literature on their impact on corpus callosum and cerebellar measurements on MRI brain scans done at term equivalent age.

## What this paper adds

1. Relatively large MRI study of corpus callosum and cerebellar measurements in preterm infants receiving corticosteroids.
2. In doses currently used, postnatal corticosteroid use did not seem to impact corpus callosum and cerebellar measurements in this cohort of extremely preterm infants.

Low‐dose systemic postnatal corticosteroids (PCS) are commonly used to aid extubation and decrease the duration of mechanical ventilator dependence in extremely preterm infants (<28 weeks' gestation) at risk of chronic lung disease (CLD).^1^ However, cranial imaging at term equivalent age (TEA) reveals that this therapy reduces volumes of various brain structures and impairs long‐term neurodevelopmental outcomes.^2^, ^3^ Long‐term effects have been demonstrated in ex‐preterm adolescents who develop smaller total brain tissue, white matter, and basal ganglia volumes than their counterparts who did not receive systemic PCS.^4^

The corpus callosum (CC) is the largest white matter tract of the brain and is responsible for the interhemispheric processing of information involving motor function, sensation, language and cognition.^5^ It is well established that low CC measurements are predictive of adverse neurocognitive and motor outcomes.^5^, ^6^, ^7^ In a small study utilising magnetic resonance imaging (MRI), nine very preterm infants exposed to low doses of systemic PCS were found to have no reduction in CC volume. This is the only study to have investigated the relationship between systemic PCS and CC size on MRI; therefore, larger and more robust studies are needed to confirm this finding.^8^ Furthermore, no studies currently quantify the relationship between cumulative corticosteroid dose and its impact on CC size.

The cerebellum also has a critical role in motor planning, cognitive function and fine‐tuning of the sensorimotor relationship through error correction.^9^ A decreased cerebellar size can result in various motor and cognitive deficits which persist long term.^10^, ^11^, ^12^ It is evidenced that administration of systemic PCS have been associated with impaired cerebellar growth and its implications on neurodevelopment.^13^, ^14^ However, this conflicts with other research demonstrating no significant impact.^15^, ^16^

The current clinical evidence recommends minimising the dose and duration of systemic PCS and limiting treatment to high‐risk cases where the neonate is still ventilator dependent after the first week of life.^17^, ^18^ As systemic PCS treatment and MRI surveillance become more prevalent in extremely preterm infants, using MRI to determine the impact of systemic PCS on CC and cerebellar size may enable the prediction of neurological impairment and therefore assist in parental counselling.^11^, ^19^

We aimed to measure CC and cerebellum sizes on TEA MRI brain scans in extremely preterm infants who received systemic PCS and compare them to those who did not receive systemic PCS. We also aimed to determine the dose‐dependent effects of systemic PCS on these outcomes.

## Methods

### Design

This retrospective cohort study involved extremely preterm infants admitted to Monash Newborn's neonatal intensive care unit (NICU) at Monash Children's Hospital, HIDDEN, Australia. Systemic PCS, oral or intravenous dexamethasone and hydrocortisone, were prescribed at the discretion of the treating team. Dosages were ascertained through scanned medication charts. MRI brain scans were accessed through the Monash Children's Hospital Imaging system. CC and cerebellar measurements were conducted by two radiologists working independently and blinded to the systemic PCS use. Monash Health Human Research Committee approved the study.

### Inclusion criteria

Participants included extremely preterm infants born under 26 weeks' gestation admitted to Monash Newborn's neonatal intensive care unit (NICU) at Monash Children's Hospital between 2016 and 2020 with an MRI brain scan conducted at TEA (routine for <26 weeks' gestational age infants in this unit).

### Exclusion criteria

Participants were excluded if they had incomplete or unretrievable data and if MRI images were degraded, hence preventing accurate measurements.

### Information collected

Demographic data were collected using patient records. Hydrocortisone was converted to standard equivalent doses of dexamethasone (dexamethasone 0.75 mg = hydrocortisone 20 mg).^20^ Standard neonatal definitions were used such as Intraventricular haemorrhage (IVH), periventricular echogenicity (PVE) and periventricular leukomalacia (PVL) identified on cranial ultrasounds. Severe IVH was defined as grade III or IV haemorrhage (Papile's classification).^21^ Chronic lung disease (CLD) was defined as any form of respiratory support or oxygen requirement beyond 36 weeks corrected gestation. Hypertensive and diabetic disorders included conditions prior to pregnancy and substance use including alcohol, cigarettes and illicit drugs. Small for gestational age (SGA) was determined using the Australian national birthweight percentiles by sex and gestational age 1998–2007.^22^ Culture‐positive bacterial sepsis included culture‐positive bloodstream, urinary tract or meningeal infections, and major surgery consisted of laparotomies, bowel resections and stoma formation.

### Outcomes

MRI brain scans were conducted on infants at TEA (at or above 35 weeks' gestational age) using a 3 T Philips machine. There were five outcomes investigated in this study. The CC was measured as corpus callosum length (CCL). CCL was also presented as a ratio against fronto‐occipital diameter (FOD) and biparietal diameter (BPD). Cerebellar measurements included the vermian height (VH) and transcerebellar diameter (TCD). CCL measurements were performed in the sagittal plane and were measured from the genu to the splenium, outer‐outer border. A coronal plane was used to measure the TCD.

### Statistical analyses

Data analysis was performed using GraphPad Prism 9.1.1 for Windows (Graphpad Software, San Diego, CA, USA) and IBM SPSS Statistics Version 28.0 for macOS (IBM Corp, Armonk, NY, USA). The number (%) was calculated for categorical data and the mean (SD) and median (IQR) were calculated for numerical data. Normality was determined with a Shapiro–Wilk test. Participants assigned to systemic PCS and no systemic PCS were compared using a *t*‐test/Mann–Whitney *U* test. Results were adjusted for significant neonatal morbidities including birthweight, discharge with home oxygen, patent ductus arteriosus ligation requiring treatment, and retinopathy of prematurity requiring treatment. A *P* value of less than 0.05 was defined as significant. The dose‐dependent relationship between cumulative corticosteroid dose, CCL, VH and TCD was determined using multiple linear regression, including dexamethasone equivalent dosing and covariates mentioned earlier.

### Ethics statement

Ethics approval was authorised by Monash Health Human Research Committee as a quality assurance project (QA/75414/MonH‐2021‐259802(v1)).

## Results

About 99 infants born under 26 weeks' gestation with MRI brain scans conducted between 34 to 46 weeks postmenstrual age were identified during the study period. Seven participants had incomplete records and were excluded. A further nine were excluded due to degraded images. A total of 83 participants were analysed; 38 exposed to systemic PCS and 45 not exposed.

Maternal and neonatal characteristics are displayed in Table 1. The median (IQR) gestational age for the unexposed group was 26 (25–26) weeks compared to 24 (24–25) weeks in the exposed group (*P* < 0.001); however, gestational age at MRI was the same between groups at 39 (38–40) weeks. Mean (SD) birthweights were statistically different at 770.6 (160) g and 664.0 (131) g in unexposed and exposed groups, respectively (*P* = 0.0015). The presence of CLD was similar with 32 (71.1%) participants in the unexposed group and 33 (86.8%) participants in the exposed group. Between unexposed and exposed groups, there were differences in median (IQR) length of first admission (122 (91–128) days vs. 125.5 (113.8–162.3) days; *P* = 0.0003), number of participants with surfactant (34 (75.6%) vs. 36 (97.4%); *P* = 0.0309), and number of participants discharge on oxygen (15 (33.3%) vs. 21 (55.4%); *P* = 0.0446). There were also differences in the number of participants with retinopathy of prematurity requiring laser (7 (15.6%) vs. 13 (34.2%); *P* = 0.0477) and patent ductus arteriosus requiring treatment (21 (46.7%) vs. 28 (73.7%); *P* = 0.0126). The mean (SD) cumulative systemic dosage in infants who received systemic PCS was 1.04 (0.80–1.49) mg during first admission.

**Table 1:** Participant demographics

	Infants who did not receive systemic PCS (*n* = 45)	Infants who received systemic PCS (*n* = 38)	*P* value
Maternal age (years)	31.4 (5.5)	31.9 (29–34.75)	0.65
Chorioamnionitis	10 (22.2)	13 (34.2)	0.22
Suspected fetal growth restriction	8 (17.8)	7 (18.9)	0.94
Hypertensive disorder in pregnancy	8 (17.8)	8 (21.1)	0.71
Diabetes in pregnancy	4 (8.9)	6 (15.8)	0.50
Abnormal CTG/Fetal compromise	19 (42.2)	16 (42.1)	0.99
Antenatal magnesium sulphate	30 (68.8)	34 (91.8)	0.87
Antenatal steroids	42 (95.5)	34 (91.8)	0.70
Substance use in pregnancy	11 (25.0)	9 (23.7)	0.94
Caesarean section	18 (40.0)	14 (36.8)	0.52
Multiple births	12 (26.7)	7 (18.4)	0.37
Gestational age (weeks)	26 (25–26)	24 (24–25)	**<0.001**
Male	22 (48.9)	24 (63.2)	0.19
Birthweight (g)	770.6 (160)	664.0 (131)	**0.001**
Small for gestational age	8 (17.8)	13 (34.2)	0.09
Apgar score 5 min	7 (6–8)	7 (6–8)	0.22
Intubation during admission	39 (86.7)	37 (97.4)	0.12
Surfactant therapy	34 (75.6)	36 (94.7)	**0.03**
Major surgery during admission	8 (17.8)	6 (15.8)	0.81
Necrotising enterocolitis requiring surgery	3 (6.7)	1 (2.6)	0.62
Chronic lung disease	32 (71.1)	33 (86.8)	0.11
Discharged on home oxygen	15 (33.3)	21 (55.4)	**0.04**
Retinopathy of prematurity requiring laser	7 (15.6)	13 (34.2)	**0.04**
Patent ductus arteriosus requiring treatment	21 (46.7)	28 (73.7)	**0.01**
Culture proven sepsis	22 (48.9)	19 (50.0)	0.92
Length of first admission (days)	112 (91–128)	125.5 (113.8–162.3)	**0.0003**
IVH on cranial ultrasound	26 (57.8)	23 (60.5)	0.80
Severe IVH on cranial ultrasound	6 (13.3)	4 (10.5)	0.75
PVE or PVL on cranial ultrasound	28 (57.8)	20 (52.6)	0.64
Cumulative steroid dosage – dexamethasone (mg)	0	1.04 (0.80–1.49)	**<0.0001**
MRI postmenstrual age (weeks)	39 (38–40)	39 (38–40)	0.92

Data presented as number (%), mean (SD), or median (IQR). *P* value determined using *Χ* ^2^, *t*‐test or Mann–Whitney *U* test as appropriate.

CTG, cardiotocography; IQR, interquartile range; IVH, intraventricular haemorrhage; MRI, magnetic resonance imaging; PVE, periventricular echodensities, PVL, periventricular leukomalacia; Systemic PCS, systemic postnatal corticosteroids.

CC and cerebellar outcomes are displayed in Table 2. These outcomes were adjusted for birthweight, retinopathy of prematurity requiring laser, patent ductus arteriosus requiring therapy, and discharge from NICU on oxygen. There was no significant difference in the adjusted mean (SE) of CCL between unexposed and exposed groups (39.5 (0.57) vs. 38.5 (0.62); *P* = 0.29). This remained insignificant when accounting for head size (CCL/FOD (0.40 (0.01) vs. 0.41 (0.01); *P* = 0.70) and CCL/BPD (0.51 (0.01) vs. 0.52 (0.01); *P* = 0.62)). Furthermore, no statistically significant differences were observed for cerebellar measurements, such as VH (24.0 (0.46) vs. 23.5 (0.51); *P* = 0.47) and TCD (49.3 (0.74) vs. 4.78 (0.82); *P* = 0.22) between the groups. The results from regression models examining the dose‐dependent effect of PCS (dexamethasone equivalent dosing) on the CC and cerebellar outcomes were insignificant.

**Table 2:** Corpus callosum and cerebellar size outcomes

	Infants who did not receive systemic PCS (*n* = 45)	Infants who received systemic PCS (*n* = 38)	Mean difference	*P* value
Corpus callosum length (mm)	39.5 (0.57)	38.5 (0.62)	−1	0.29
Corpus callosum length/fronto‐occipital diameter	0.40 (0.01)	0.41 (0.01)	+0.01	0.70
Corpus callosum length/biparietal diameter	0.51 (0.01)	0.52 (0.01)	+0.01	0.62
Vermian height (mm)	24.0 (0.46)	23.5 (0.51)	−0.5	0.47
Transcerebellar diameter (mm)	49.3 (0.74)	47.8 (0.82)	−1.5	0.22

Data presented as mean (SEM).

Covariates appearing in the model: Birthweight (g), patent ductus arteriosus requiring treatment, retinopathy of prematurity requiring treatment, discharge on home oxygen.

Systemic PCS, systemic postnatal corticosteroids.

## Discussion

Our study compared CC and cerebellum measurements on TEA MRIs in extremely premature infants who were exposed to systemic PCS to those without exposure. We found no significant differences in CC or cerebellar measurements between infants exposed to systemic PCS compared to infants who were not exposed to systemic PCS. Additionally, we found no dose‐dependent relationship between systemic PCS and CC or cerebellar measurements in extremely preterm infants exposed to systemic PCS.

Reduced CC and cerebellar sizes are well‐established predictive variables for adverse neurocognitive and motor outcomes.^5^, ^6^, ^7^, ^10^, ^11^, ^12^ Our study has demonstrated no association between PCS and CC and cerebellar size, and suggests that the use of systemic PCS at current recommended doses does not impact the growth of these vital brain structures in extremely preterm infants and may not affect their neurocognitive and motor outcomes. However, more and larger studies are required to confirm this finding. Notably, this study has analysed the largest number of infants exposed to systemic PCS and its impact on CC size via MRI to date.

The cumulative systemic PCS dose over the course of treatment used was a mean (SD) of 1.082 (0.69) mg of dexamethasone equivalent, which is relatively low. In comparison, randomised trials assessing the efficacy and safety of systemic PCS have used doses anywhere between 0.89 and 14 mg/kg of dexamethasone. Clinicians being aware of the detrimental effects of high‐dose PCS tend to be cautious and use PCS at the lower end of the spectrum, as evident in our study, which may not have a significant effect on the brain. Our study did not show any dose‐dependent relationship between systemic PCS and CC and cerebellar measurements, but we certainly cannot rule out this relationship with dosing outside the range used in this study.

Infants were administered systemic PCS as per clinical protocol; after the first week of post‐natal life and if ventilator dependent. The effect of systemic PCS on death or cerebral palsy is greatly influenced by the timing of initiation and the risk of developing bronchopulmonary dysplasia.^17^, ^18^ These risks are higher if PCS is used earlier or when the risk of developing CLD is not substantially high. We can presumably extrapolate the balance of risk versus benefit with systemic PCS in relation to brain measurements and perhaps deduce that the brain measurements may not be affected when the PCS use is selective.

The impact of systemic PCS on CC size via MRI has only been achieved in one other study aiming to identify perinatal influences on the CC.^8^ In this study of 106 preterm infants, there was a statistically significant change in CC shape, but no reduction in CC segmental areas.^8^ Our CC findings align with this conclusion. Notably, there were only nine infants exposed to systemic PCS and dose‐dependence was not evaluated. Evidently, there is a deficit of comprehensive research in this field and marked limitations in generalising conclusions made from nine infants. The lack of larger studies in current literature highlights our study's valuable contribution having analysed the largest number of infants exposed to systemic PCS and its impact on CC size via MRI to date.

There is currently mixed evidence about the impact of systemic PCS on cerebellar size as measured by MRI. A study of 41 infants (11 exposed to systemic PCS vs. 30 not exposed) identified an association between systemic PCS and smaller cerebellar volumes on TEA MRIs in extremely low birthweight infants.^2^ However, the mean dexamethasone dosage was 2.8 mg/kg with a range of 1.2–5.8 mg/kg, substantially larger dosages than our study. These statistically significant findings were further supported by another study of 132 preterm infants (42 exposed to systemic PCS vs. 90 not exposed) which demonstrated an association between post‐natal hydrocortisone and dexamethasone use and decreased cerebellar volume on MRI.^13^ The median total dexamethasone exposure was 0.89 mg and hydrocortisone was 11.58 mg. In this study, both steroids were studied independently instead of assessing cumulative corticosteroid effect. As our study converted hydrocortisone into dexamethasone equivalent doses, we were able to assess relationships based on cumulative corticosteroid exposure which may explain the difference in our conclusions. Concordant with our study, no dose‐dependent effects were found at dosages used in current clinical treatment. In contrast to these significant findings, a 2019 study utilising PCS as a covariate demonstrated no cerebellar volume differences associated with PCS.^15^ This conclusion is corroborated by another study revealing no difference in cerebellar volume associated with hydrocortisone use.^16^ Our study aligns with these findings further evidencing no significant impact of systemic PCS on cerebellar size at clinically utilised dosages.

Despite our study being large relative to current literature, there were limitations associated with its small sample size and single centre design. There are also limitations in the outcomes and measurement techniques we elected to use. We did not include neurodevelopmental follow‐up in this study, which limits the clinical applicability of our findings. However, our surrogate outcomes were objective, blinded and have been shown to have good correlation with functional outcomes. Furthermore, in current literature, the structure of the CC has been commonly quantified as segmental cross‐sectional area, fractional anisotropy or diffusivity.^8^, ^23^ Cerebellar size has often been assessed through segmental volumetric analysis measured by semi‐automated 3D reconstruction.^2^, ^13^, ^15^, ^16^ We utilised CCL, VH and TCD due to the ease of simple linear measurements when considering an outcome's clinical implications as practical and reproducible criterion in MRI assessment tools for brain injury. We recognise that this representation is a simplification that precludes the detailed detection of impairments to segmental volume, density and rates of growth. Despite this, linear measurements are evidenced to clinically correlate to neurodevelopmental outcome on MRIs.^24^ Ratios involving head sizes were included to account for the size of the infant at the time of MRI brain scan. Additionally, by converting hydrocortisone to dexamethasone equivalent dosages, we have not considered the potential for their distinct effects on brain structures given their mechanistic differences in action.^16^ Subgroup analysis was not conducted due to our small group sizes which may result in false associations. Future studies with larger cohort sizes would benefit from determining both cumulative and independent impacts.

Baseline differences in participant demographics between groups were significant due to the observational nature of our data, in which infants who require systemic PCS would inherently be smaller with more comorbidities. Given this is the population who will be likely to require this therapy, we have aptly demonstrated that our current clinical use of systemic PCS does not impact the growth of these structures in the most likely context. In attempt to isolate the true effect of systemic PCS on these brain structures, we adjusted for differences in these post‐natal variables. We have recognised that this can create overadjustment bias which impacts generalisability to the population of interest.

## Conclusions

In this retrospective single‐centre study comprising of extremely preterm infants born under 26 weeks' gestation, we did not find an association between systemic PCS exposure and CC and cerebellar size. Additionally, we did not find dose‐dependent relationships between these outcomes and systemic PCS at low dosages. Larger studies are required to determine the association between hydrocortisone and dexamethasone independently and include long‐term neurodevelopmental follow‐up of participants.

## Supporting information


**Table S1.** Multiple linear regression analysis demonstrating no dose‐dependent relationship between postnatal steroids, assessed as dexamethasone equivalent dosing, and corpus callosum length (*P* = 0.577)
**Table S2.** Multiple linear regression analysis demonstrating no dose‐dependent relationship between postnatal steroids, assessed as dexamethasone equivalent dosing, and corpus callosum length/fronto‐occipital diameter (*P* = 0.724)
**Table S3.** Multiple linear regression analysis demonstrating no dose‐dependent relationship between postnatal steroids, assessed as dexamethasone equivalent dosing, and corpus callosum length/biparietal diameter (*P* = 0.828)
**Table S4.** Multiple linear regression analysis demonstrating no dose‐dependent relationship between postnatal steroids, assessed as dexamethasone equivalent dosing, and vermis height (*P* = 0.155)
**Table S5.** Multiple linear regression analysis demonstrating no dose‐dependent relationship between postnatal steroids, assessed as dexamethasone equivalent dosing, and transcerebellar diameter (*P* = 0.291)
