# Supplementary material for: Systemic postnatal corticosteroids and magnetic resonance imaging measurements of corpus callosum and cerebellum of extremely preterm infants
Source: J Paediatr Child Health. 2022 Nov 20;59(2):282–7. doi: 10.1111/jpc.16286 (PMC10098787; doi:10.1111/jpc.16286)
Supplement: Supplementary file 1 — Table S1. Multiple linear regression analysis demonstrating no dose‐dependent relationship between postnatal steroids, assessed as dexamethasone equivalent dosing, and corpus callosum length (P = 0.577) Table S2. Multiple linear regression analysis demonstrating no dose‐dependent relationship between postnatal steroids, assessed as dexamethasone equivalent dosing, and corpus callosum length/fronto‐occipital diameter (P = 0.724) Table S3. Multiple linear regression analysis demonstrating no dose‐dependent relationship between postnatal steroids, assessed as dexamethasone equivalent dosing, and corpus callosum length/biparietal diameter (P = 0.828) Table S4. Multiple linear regression analysis demonstrating no dose‐dependent relationship between postnatal steroids, assessed as dexamethasone equivalent dosing, and vermis height (P = 0.155) Table S5. Multiple linear regression analysis demonstrating no dose‐dependent relationship between postnatal steroids, assessed as dexamethasone equivalent dosing, and transcerebellar diameter (P = 0.291) [file JPC-59-282-s001.docx]

**Supporting Information**

e-Table 1: Multiple linear regression analysis demonstrating no dose-dependent relationship between postnatal steroids, assessed as dexamethasone equivalent dosing, and **Corpus Callosum length** (P=0.577)

| **Coefficients** | | | | | | | | |
| --- | --- | --- | --- | --- | --- | --- | --- | --- |
| Model | | Unstandardized Coefficients | | Standardized Coefficients | t | Sig. | 95.0% Confidence Interval for B | |
|  |  | B | Std. Error | Beta |  |  | Lower Bound | Upper Bound |
| 1 | (Constant) | 32.814 | 2.271 |  | 14.452 | .000 | 28.292 | 37.335 |
|  | Birth weight (g) | .009 | .003 | .375 | 3.347 | .001 | .004 | .015 |
|  | Dexamethasone equivalent dose (mg) | -.302 | .539 | -.064 | -.561 | .577 | -1.377 | .772 |
|  | Discharged from NICU on Oxygen | -1.002 | .840 | -.128 | -1.192 | .237 | -2.674 | .671 |
|  | ROP requiring laser | .258 | 1.035 | .028 | .249 | .804 | -1.803 | 2.318 |
|  | PDA requiring Rx | -.050 | .898 | -.006 | -.056 | .955 | -1.838 | 1.737 |
|  | | | | | | | | |

e-Table 2: Multiple linear regression analysis demonstrating no dose-dependent relationship between postnatal steroids, assessed as dexamethasone equivalent dosing, and **Corpus Callosum Length/Fronto-occipital diameter** (P=0.724)

| **Coefficients** | | | | | | | | |
| --- | --- | --- | --- | --- | --- | --- | --- | --- |
| Model | | Unstandardized Coefficients | | Standardized Coefficients | t | Sig. | 95.0% Confidence Interval for B | |
|  |  | B | Std. Error | Beta |  |  | Lower Bound | Upper Bound |
| 1 | (Constant) | .466 | .047 |  | 9.880 | .000 | .372 | .560 |
|  | Birth weight (g) | -7.441E-5 | .000 | -.154 | -1.275 | .206 | .000 | .000 |
|  | Dexamethasone equivalent dose (mg) | .004 | .011 | .043 | .354 | .724 | -.018 | .026 |
|  | Discharged from NICU on Oxygen | .008 | .017 | .053 | .459 | .648 | -.027 | .043 |
|  | ROP requiring laser | .005 | .022 | .026 | .211 | .833 | -.038 | .047 |
|  | PDA requiring Rx | -.029 | .019 | -.187 | -1.533 | .129 | -.066 | .009 |
| e-Table 3: Multiple linear regression analysis demonstrating no dose-dependent relationship between postnatal steroids, assessed as dexamethasone equivalent dosing, and **Corpus Callosum Length/Biparietal diameter** (P=0.828)   \| **Coefficients** \| \| \| \| \| \| \| \| \| \| --- \| --- \| --- \| --- \| --- \| --- \| --- \| --- \| --- \| \| Model \| \| Unstandardized Coefficients \| \| Standardized Coefficients \| t \| Sig. \| 95.0% Confidence Interval for B \| \| \| B \| Std. Error \| Beta \| Lower Bound \| Upper Bound \| \| 1 \| (Constant) \| .445 \| .050 \|  \| 8.989 \| .000 \| .347 \| .544 \| \| Birth weight (g) \| 9.942E-5 \| .000 \| .195 \| 1.622 \| .109 \| .000 \| .000 \| \| Dexamethasone equivalent dose (mg) \| .003 \| .012 \| .026 \| .217 \| .828 \| -.021 \| .026 \| \| Discharged from NICU on Oxygen \| -.023 \| .018 \| -.146 \| -1.268 \| .209 \| -.060 \| .013 \| \| ROP requiring laser \| .018 \| .023 \| .097 \| .790 \| .432 \| -.027 \| .063 \| \| PDA requiring Rx \| .005 \| .020 \| .029 \| .236 \| .814 \| -.034 \| .044 \| | | | | | | | | |

e-Table 4: Multiple linear regression analysis demonstrating no dose-dependent relationship between postnatal steroids, assessed as dexamethasone equivalent dosing, and **Vermis Height** (P=0.155)

| **Coefficients** | | | | | | | | |
| --- | --- | --- | --- | --- | --- | --- | --- | --- |
| Model | | Unstandardized Coefficients | | Standardized Coefficients | t | Sig. | 95.0% Confidence Interval for B | |
|  |  | B | Std. Error | Beta |  |  | Lower Bound | Upper Bound |
| 1 | (Constant) | 21.478 | 1.816 |  | 11.829 | .000 | 17.863 | 25.094 |
|  | Birth weight (g) | .003 | .002 | .167 | 1.388 | .169 | -.001 | .008 |
|  | Dexamethasone equivalent dose (mg) | .620 | .431 | .175 | 1.437 | .155 | -.239 | 1.479 |
|  | Discharged from NICU on Oxygen | -.650 | .672 | -.111 | -.968 | .336 | -1.988 | .688 |
|  | ROP requiring laser | .873 | .827 | .129 | 1.055 | .295 | -.775 | 2.520 |
|  | PDA requiring Rx | -.334 | .718 | -.057 | -.466 | .643 | -1.764 | 1.095 |
|  | | | | | | | | |

e-Table 5: Multiple linear regression analysis demonstrating no dose-dependent relationship between postnatal steroids, assessed as dexamethasone equivalent dosing, and **Transcerebellar Diameter** (P=0.291)

| **Coefficients** | | | | | | | | |
| --- | --- | --- | --- | --- | --- | --- | --- | --- |
| Model | | Unstandardized Coefficients | | Standardized Coefficients | t | Sig. | 95.0% Confidence Interval for B | |
|  |  | B | Std. Error | Beta |  |  | Lower Bound | Upper Bound |
| 1 | (Constant) | 44.700 | 2.961 |  | 15.099 | .000 | 38.805 | 50.596 |
|  | Birth weight (g) | .006 | .004 | .207 | 1.734 | .087 | -.001 | .014 |
|  | Dexamethasone equivalent dose (mg) | .748 | .703 | .129 | 1.063 | .291 | -.653 | 2.149 |
|  | Discharged from NICU on Oxygen | -.691 | 1.095 | -.072 | -.631 | .530 | -2.872 | 1.490 |
|  | ROP requiring laser | 1.239 | 1.349 | .112 | .919 | .361 | -1.447 | 3.926 |
|  | PDA requiring Rx | -1.833 | 1.170 | -.190 | -1.567 | .121 | -4.164 | .497 |
|  | | | | | | | | |
